# Involving parents of children treated for cancer in Sweden as public contributors to inform the design and conduct of an evaluation of internet-administered self-help for parents of children treated for cancer: a protocol

**DOI:** 10.1186/s40900-023-00532-4

**Published:** 2024-01-02

**Authors:** Joanne Woodford, Christina Reuther, Johan Lars Ljungberg, Louise von Essen

**Affiliations:** Healthcare Sciences and E-Health, Department of Women’s and Children’s Health, Uppsala University, Dag Hammarskjölds Väg 14B, 751 05 Uppsala, Sweden

**Keywords:** Childhood cancer, e-Health, Intervention, Parents, Public contribution, Public involvement

## Abstract

**Introduction:**

Public contribution in research can facilitate the design and conduct of meaningful research, resulting in feasible and sustainable solutions to healthcare challenges. However, the evidence concerning the acceptability, feasibility, and impact of public contribution in research is limited. We will embed a mixed-method examination of public contribution activities into the CHANGE trial. The overall aim of the CHANGE trial is to evaluate the efficacy and cost-effectiveness of an internet-administered, guided, low-intensity cognitive behavioral therapy-based self-help intervention (EJDeR) plus treatment as usual (TAU) versus TAU for symptoms of depression and/or Generalized Anxiety Disorder in a superiority randomized controlled trial with an internal pilot phase. In this protocol we describe how we aim to: (1) involve parents of children treated for cancer in the managing and undertaking, analysis and interpretation, and dissemination phases of the CHANGE trial; and (2) examine the acceptability, feasibility, and perceived impact of Parent Advisory Board contribution to the trial from the perspective of board members and public contribution coordinators.

**Methods:**

We will recruit around six parents of children treated for cancer to the Parent Advisory Board. Board members will contribute throughout the trial during online workshops and steering group meetings. An impact log will be used during workshops to record activities and examine the perceived impact of activities according to board members and public contribution coordinators, including anticipated and unanticipated changes to the research process and potential benefits and harms. Activities will be reported using the Guidance for Reporting Involvement of Patients and the Public checklist. We will conduct semi-structured interviews with board members and public contribution coordinators 6 months after the board is established and at the end of the trial to examine the acceptability, feasibility, and perceived impact of public contribution activities. We will also conduct interviews with board members and public contribution coordinators who withdraw participation. Findings will be reported in accordance with the Standards for Reporting Qualitative Research checklist.

**Discussion:**

We hope adding public contribution to the CHANGE trial will provide guidance on how to embed public contribution in research and add to the evidence base concerning the impact of public contribution.

**Supplementary Information:**

The online version contains supplementary material available at 10.1186/s40900-023-00532-4.

## Abstract (plain language summary)

Involving the public in research can help improve research. However, we do not know so much about what impact it has. Here, we describe how we will involve parents of children treated for cancer in the CHANGE trial and how we will examine the impact of their contribution.

Even years after end of treatment parents can experience difficult emotions, such as depression and anxiety. We have therefore developed an internet-administered self-help intervention called EJDeR for parents of children treated for cancer. In the CHANGE trial we will evaluate whether EJDeR reduces parent’s depression and anxiety.

We will recruit around six parents to a Parent Advisory Board. Board members will help us to: (1) manage and undertake the CHANGE trial including designing trial procedures e.g., participant-facing material and interview guides; and to steer the trial e.g., discuss trial progress and produce research updates; (2) interpret findings; and (3) plan how to communicate findings to parents and the surrounding community.

Board members will participate in online workshops. We will record all activities and whether, and if so how, activities are perceived to impact on the CHANGE trial. Board members will also participate in steering meetings with members of our research team. We will interview board members and public contribution coordinators about their experiences working with us and contributing to the CHANGE trial. We hope this approach will help us and other researchers to understand the potential impact of public contribution on research.

**Supplementary Information:**

The online version contains supplementary material available at 10.1186/s40900-023-00532-4.

## Introduction

Public contribution in research is widely recognized and recommended by researchers, funders, and policy makers [1, 2]. Recognition stems from identified benefits to healthcare research, such as enhancing research quality, relevance, and usefulness [3–6] e.g., by focusing on the needs and preferences of the public, i.e., the target population. Specific benefits include developing and prioritizing research questions of importance to the public, informing the development of acceptable and feasible recruitment strategies; enhancing the accessibility and user-friendliness of participant-facing materials; facilitating data collection [3–8]; enhancing the rigor and validity of data analysis and interpretation [3, 9]; and widening dissemination [10]. Public contribution is also associated with improving knowledge of research among and empowering public contributors [7].

Public contributors’ lived experience of a particular health condition is what is unique about their contribution, and they may also bring occupational knowledge and skills, and an “outsider” perspective to the research team [11]. With public contribution, the public is involved as partners in research i.e., as a member of an advisory group/advisory board, or steering committee [5] and asked to bring their views, knowledge, and experiences to the table. Public contribution can include but is not limited to, working with research funders to prioritize research, offering advice, and working alongside a research team. The use of Public Advisory Groups in clinical research e.g., collaborating with trial teams to formulate research questions, plan trial methods and procedures, collect, analyze, and report data, and disseminate findings is anticipated to become standard practice [12].

Best practice guidelines exist for planning and implementing Public Advisory Groups, however, these have been developed for pharmaceutical and biotechnology companies [13] and may not be transferable to all clinical trial settings, for example, of complex healthcare interventions. In addition, despite an increasing number of reviews highlighting the impact and benefits of public contribution [3–7], the evidence base is limited by poor and inconsistent reporting [3, 14, 15]. There is also a lack of standardized guidance on how to evaluate public contribution [16]. The existing evidence base is criticized for lack of scientific rigor concerning evaluation tools [17] with existing tools failing to capture the complexity and possible outcomes of public contribution [18]. Further, public contribution in Nordic healthcare research remains in its infancy in comparison with other European countries such as the United Kingdom (UK) [19]. Therefore, there is a need especially in the Nordic countries [19, 20] to enhance researcher understanding regarding how to best implement public contribution in research [6] and improve the evidence base concerning its potential impact.

### Context: the CHANGE trial

Parents are the primary source of support for children with cancer and many are actively involved in the child’s care, even years after treatment. Sub-groups report symptoms of and depression (14%) and anxiety (20%) [21], problems with depression and/or anxiety [22], productivity losses [23–25], and/or an unmet need for psychological support [26] after end of treatment. In spite of this, there is a lack of evidence-based interventions tailored to the population, with psychological needs commonly unmet. Parents report barriers to seeking support such as lack of time, guilt, and putting the child’s health first [26–30]. Innovations to increase access to evidence-based psychological interventions are implemented worldwide in the form of low-intensity cognitive behavioral therapy (LICBT), where evidence-based cognitive behavioral therapy (CBT) techniques are delivered in a self-help format e.g., via written workbooks and health technology (i.e., internet-administered, smartphone applications, and audio-books) [31]. An internet-administered, guided, LICBT based self-help intervention may represent a solution to provide support to parents of children treated for cancer.

Informed by the 2021 UK Medical Research Council (MRC) complex interventions framework [32], we alongside fathers and mothers of children treated for cancer i.e., parent research partners (PRPs) following Phase I of the framework developed the internet-administered, guided, LICBT based self-help intervention, EJDeR (Swedish acronym for int**E**rnetbaserad s**J**älvhjälp för föräl**D**rar till barn som avslutat en behandling mot canc**eR**) [31, 33–37]. EJDeR is tailored towards depression and Generalized Anxiety Disorder (GAD) and is delivered via the U-CARE Platform, an in-house web-based platform, designed to deliver internet-administered complex healthcare interventions and support the digital execution of study procedures, e.g., online randomization, informed consent, and data collection. A detailed description of EJDeR has been published [36]. Based on PRPs’ preferences we developed study procedures for a superiority randomized controlled trial (RCT) to evaluate the clinical efficacy and cost-effectiveness of EJDeR plus treatment as usual (TAU) versus TAU. Examples of procedures include the mode of follow-up reminders being based on preference i.e., via telephone, SMS, or e-mail, personalization of automated reminders, and study newsletter scheduling. Following Phase II of the 2021 MRC framework [32] we undertook the single-arm uncontrolled feasibility trial ENGAGE [38]. A priori progression criteria were set [39] and results showed that study procedures and EJDeR are acceptable and feasible [38]. Next, we will evaluate the efficacy and cost-effectiveness of EJDeR plus TAU vs TAU for symptoms of depression and/or GAD in a superiority RCT with an internal pilot phase (the CHANGE trial). As the first step we will establish a Parent Advisory Board (PAB) who will contribute to the CHANGE trial [12, 13]. Public contribution coordinators, one internal to our research team and one external, will facilitate the PABs’ contribution to the CHANGE trial.

### Aims

The aims are to: (1) involve parents of children treated for cancer in the managing and undertaking, analysis and interpretation, and dissemination phases of the CHANGE trial [12, 13]; and (2) examine the acceptability, feasibility, and perceived impact of PAB contribution to the trial from the perspective of board members and public contribution coordinators.

## Methods and analysis

We have developed this protocol in accordance with the Guidance for Reporting Involvement of Patients and the Public Checklist (GRIPP2; see Additional file 1) [40].

### Public contribution framework

Public contribution will be embedded into the CHANGE trial following the UK National Institute for Health Research (NIHR) framework [41].

### Setting

The research will be conducted from Uppsala University, Sweden. We intend to hold eight online workshops with the PAB each anticipated to last 4 h, to inform how to evaluate EJDeR, interpret trial findings, and plan how to communicate findings to parents and the surrounding community. The length and frequency of workshops may change according to PAB member preference. We will also hold bi-monthly meetings with PAB members and our research team, anticipated to last approximately 1 h, to steer the CHANGE trial (e.g., discuss trial progress, assist in problem-solving potential challenges, and produce research updates in collaboration with our research team (e.g., trial newsletters)). We anticipate the total duration of the CHANGE trial, and public contribution activities, to be approximately 3 years.

### Public contribution coordinators

Informed by preferences voiced by parents who contributed to research informing the development of EJDeR and study procedures [34], public contribution activities will be coordinated and facilitated by public contribution coordinators both internal and external to the research team. The public contribution coordinators will be trained and provided with continuous supervision on public contribution by the principal investigator (PI), Louise von Essen (LvE), and co-investigator Joanne Woodford (JW) who both have experience of public contribution in healthcare research. The public contribution coordinators will arrange and facilitate PAB workshops and meetings, liaise with the research team, and support PAB members. They will also be responsible for maintaining contact with PAB members e.g,. over the telephone, e-mail, online communication platform, or SMS communication, and distributing any necessary materials.

### PAB members

PAB members will be parents, of at least 18 years of age, whose child was diagnosed with cancer when 0–18 years and has completed treatment 3 months to 5 years previously. They will be able to speak, read, and understand Swedish.

### Recruitment of PAB members

PAB members will be recruited via advertisements posted on websites of childhood cancer and carer organizations, interest groups, and social media sites.

#### Initial interest and consent

Those interested in being a board member will be encouraged to contact our research team via e-mail, telephone, or SMS. Following guidelines for public contribution in research [41, 42] those who contact us will be contacted by a member in our research team to: (1) explain the purpose of the PAB; (2) explore any barriers to participation in the PAB (for example working hours or childcare demands) and how these barriers might be overcome; (3) explain that PAB members will participate in online workshops, steering group meetings and semi-structured interviews; and (4) inform about the reimbursement for contribution: 1000 SEK (≈ EURO 85) for each workshop and 250 SEK (≈ EURO 22) for each steering meeting and interview.

When talking to interested parents we will also inform them that we wish to form a PAB with varied backgrounds regarding age, gender, educational level, and location lived in. We will invite parents to a recruitment meeting (≈ 2 h) on an online communication platform with the public contribution coordinators, the PI and other parents who have expressed an interest of being a PAB member. Those still interested will be sent written information about what it means to be a member of the PAB, a background questionnaire (the parent’s age, educational background, gender, location lived in, and relationship status, and the child’s age, cancer diagnosis, and started and end date of cancer treatment), a consent form to workshops and steering group meetings, and a pre-stamped envelope. Those who want to be a member of the PAB will be asked to return the completed questionnaire and signed consent form to our research team in the pre-stamped envelope within 2 weeks. Around 10 parents who have provided consent will be invited to the recruitment meeting.

#### Recruitment meeting

During the recruitment meeting, we will explain the purpose of the PAB, the structure and frequency of workshops, meetings, and semi-structured interviews. Informed by research highlighting the importance of clarifying expectations and roles when recruiting into public advisory boards [43] we will explore expectations and motivations for wishing to be a PAB member. After the meeting, the public contribution coordinator and PI in collaboration with members of our research team will invite around six parents to the PAB. Invitations will be based on wishing to form a PAB with varied backgrounds as well as parents’ expectations and motivations [43]. Decisions with an explanation as to why a parent is invited to the PAB or not will be provided by the public contribution coordinators and/or the PI during an individual meeting with each parent. Given the PAB will work alongside the research team throughout the research lifecycle of the CHANGE trial, we anticipate some members will withdraw from the PAB.

#### Withdrawal

To maintain a sufficient number we will replace PAB members who withdraw. This will be done by conducting a new group recruitment meeting or holding individual recruitment meetings, dependent on factors such as the number of PAB members to be replaced, the number of parents interested in joining the PAB, and project resources. We will invite all PAB members and public contribution coordinators who withdraw to a semi-structured interview (see data collection).

### Workshops and meetings

We intend to hold eight PAB workshops to inform how to manage and undertake the CHANGE trial, interpret findings, and plan how to communicate results to parents and the surrounding community. We will also conduct bi-monthly steering meetings with PAB members and members of our research team to steer the trial. PAB members will have the option to attend meetings online or in real life at Uppsala University. We will provide options for telephone, e-mail, online communication platform, or SMS communication with PAB members where needed, for example, if unable to attend a scheduled workshop or meeting. PAB workshops will be held online and we will follow recommendations for conducting online public contribution activities [44]. For example, one public contribution coordinator will adopt the role of meeting chair and be responsible for ensuring active inclusion of all PAB members in discussions, encourage camera sharing, and schedule time for breaks to facilitate more informal social interactions.

Due to limited flexibility i.e., project timeline to rearrange workshops in situations where multiple PAB members are unable to attend, the minimum attendance necessary to run a scheduled workshop will be set at three members. PAB members unable to attend scheduled workshops may be offered individual meetings dependent on project need and resources.

If a PAB member fails to attend multiple PAB workshops and/or meetings, a public contribution coordinator will attempt to reach the person via e.g., telephone to explore reasons for lack of attendance and whether the PAB member is still able, motivated, and willing to be a public contributor, and whether to stop their involvement.

### Public contribution activities

Activities will include: (1) managing and undertaking the CHANGE trial (a) designing trial procedures (e.g., recruitment and retention of potential study participants, development of participant-facing material (i.e., participant invitation letters, study information sheets, and consent forms), and the identification of appropriate outcome measures and measurements and data collection procedures) [45, 46] and (b) steering the trial (e.g., discussing trial progress and assisting with problem-solving potential challenges, and producing research updates (e.g., study newsletters)) [12, 13]; (2) analysis and interpretation: interpretation and sense-making of research findings [47, 48]; and (3) dissemination outside of academia: development of plain language summaries and strategies for local dissemination of findings, and exhibitions [10, 49]. We anticipate types of contribution and participation (e.g., consultation where contributors are asked for their opinion to inform decision-making by the research team, or collaboration whereby contributors and the research team share aspects of decision-making [42]) to change, dependent on the research phase. During the analysis and interpretation phase, we anticipate contribution to be at the consultation level, whereby PAB members are presented with preliminary findings and asked for feedback, rather than the collaboration level where both PAB members and the research team analyze the data. However, at the dissemination phase, collaboration is expected, whereby PAB members and the research team will co-produce a plan for dissemination outside of academia. PAB activities will be mapped onto a Participation Matrix [50] adapted to report type of contribution for different research activities [51]. Ability to make decisions concerning certain phases (e.g., study design) will be limited due to funder requirements and scientific considerations. If PAB members make suggestions that are not possible to implement there will be transparency in the decision-making process, i.e., PAB members will be clearly informed as to why suggestions were not implemented [52].

Preliminary PAB workshop topics are outlined in Table 1. Suggested topics for the analysis and interpretation and dissemination phases are outlined in less detail than for the managing and undertaking phases given the exact content may be subject to more change according to PAB contribution than the first phase. Prior to each PAB workshop, an agenda will be prepared by public contribution coordinators in line with the workshop topic and distributed to PAB members. Materials to be discussed (e.g., participant-facing material such as participant invitation letters, participant information sheets, and consent forms) may also be distributed. PAB members will be able to add their own agenda items. Meeting agendas will also be prepared and distributed to PAB members in advance of bi-monthly trial steering meetings with members of our research team.

**Table 1:** Preliminary Parent Advisory Board (PAB) workshop topics to inform the managing and undertaking, analysis and interpretation, and dissemination phases of the CHANGE trial

Workshop	Main topic	Subtopics
*Phase: Managing and undertaking*		
1	Introduction to the CHANGE trial and the PAB	Introduction to the CHANGE trial Introduction to public contribution in research Expectation setting PAB preference concerning PAB structure PAB preference concerning PAB location PAB preference concerning workshop structure Clarifying roles and responsibilities
2	Recruitment, retention, participant information sheets, participant invitations	Recruitment and retention procedures Recruitment advertisements Written material Participant invitation letter Participant information sheet Paper reply slip to register interest Paper reply slip to opt-out Reasons for non-participation questionnaire
3	Outcome measures and measurements	Clinical outcomes and measurements Health economic outcomes and measurements Sociodemographic and clinical factors and measurements
4	Interview material	Reasons for non-participation topic guide Post-treatment semi-structured interview topic guide
	Ethical considerations	Privacy Identity protection Sensitive information and how to mitigate them
*Phase: Data analysis and interpretation*		
5	Reasons for non-participation	Analysis of reasons for non-participation in the CHANGE trial and sense-making and interpretation of results
6	CHANGE trial-results—clinical efficacy and cost effectiveness	Analysis of results from the CHANGE trial and sense-making and interpretation of results
7	Acceptability of EJDeR	Analysis of results regarding the acceptability of EJDeR and sense-making and interpretation of results
*Phase: Dissemination*		
8	Dissemination plan	Development of a dissemination plan e.g., outside of academia
	Plain language summaries	Development of plain language summaries of results in Swedish and English

Given we aim to explore the acceptability and feasibility of public contribution, we will be open and flexible to adapting public contribution activities in collaboration with PAB members.

### Data collection

#### Workshops

During and immediately after each workshop public contribution coordinators will note PAB activities in an impact log [53]. Impact logs include: (1) the date and time of the workshop; (2) the location of the workshop; (3) the type of activity; (4) who took part; (5) who were absent; (6) who were absent with apologies; (7) ideas and suggestions from PAB members; (8) perceived impact e.g., changes and adaptations to the research process and potential benefits and harms due to ideas and suggestions from PAB members; and (9) next steps. The public contribution coordinators will present the content in the impact log to PAB members present at the beginning of each subsequent PAB workshop to gain feedback and check accuracy. Discussions at workshops will be audio-recorded with informed consent to ensure the accuracy of logged activities, audio recordings will not be transcribed.

#### Steering group meetings

During and immediately after each meeting the public contribution coordinators will record decisions and discussions in meeting protocols.

#### Semi-structured interviews

We will invite all PAB members to a semi-structured interview via telephone or an online platform 6 months after the PAB has been formed and at the end of the CHANGE trial to explore the acceptability, feasibility, and perceived impact of PAB activities. Members of the research team not participating in PAB activities will conduct interviews with the PAB. Public contribution coordinators will be invited to a semi-structured interview in person to explore the acceptability, feasibility, and perceived impact of PAB activities. A researcher external to the research team will conduct the interviews. The interview guides, informed by previous research [53], will explore the perceived: purpose, importance, and impact of the PAB; barriers and facilitators to public contribution e.g., difficulties or challenges experienced; benefits of working with the PAB; and suggested improvements to the PAB activities.

Each interview will be audio-recorded with informed consent and transcribed verbatim. PAB members will be informed about the interview at a workshop, meeting, or over the telephone, approximately 4 weeks prior to the respective interview. Thereafter PAB members will be sent a consent form and a pre-stamped envelope. Those interested will be asked to return the signed consent form in the pre-stamped envelope to the research team. Public contribution coordinators will also be provided with oral information and asked for consent prior to the interview.

PAB members who withdraw before the end of the CHANGE trial will be invited into a semi-structured interview. They will be sent a consent form and a pre-stamped envelope. Those interested will be asked to return the signed consent form in the pre-stamped envelope to the research team. PAB members will not be required to have attended any workshops or meetings to be invited to participate in the interviews.

Public contribution coordinators who stop working as a public contribution coordinator before the end of the CHANGE trial will be invited into a semi-structured interview. They will be provided with oral information and asked for consent prior to the interview.

### Data analysis

#### Impact logs and protocols from meetings

Two members of the research team not involved in PAB activities will individually read impact logs, with impacts e.g., anticipated and unanticipated changes to the research process and potential benefits and harms extracted and summarized. Ideas and suggestions arising from workshops and meetings will be categorized (i.e., recruitment procedures, retention procedures, participant-facing material, clinical outcomes and measurements, dissemination). The number of ideas and suggestions made by PAB members and the number implemented will be counted for each category. The percentage of ideas and suggestions implemented for each category will be calculated and reported. Reasons for not implementing ideas and suggestions will be provided where necessary [54].

#### Transcripts of semi-structured interviews

Two members of the research team not involved in PAB activities will individually code transcripts and analyze the data using manifest content analysis. Categorization of codes into categories and subcategories will be performed individually and subsequently discussed in analysis workshops with members of the research team [55]. We will establish trustworthiness [56] via disconfirming case analysis (i.e., actively searching for cases that do not fit emerging categories and subcategories); triangulation of investigators (i.e., more than one member of the research team involved in analysis and discussion in data analysis workshops); peer examination (i.e., research procedures and findings discussed with members of the research team not involved in analysis or PAB activities); and audit trail (i.e., data collection instruments, raw data, data analysis process, procedures, and decision making).

### Reporting

We will report PAB activities and perceived impact in accordance with the GRIPP2 checklist [40] and qualitative results in accordance with the Standards for Reporting Qualitative Research checklist (SRQR) [57].

### Ethical considerations

Ethical approval was obtained from the Swedish Ethical Review Authority (Dnr:2023-06850-01). We will conduct all activities in accordance with the Declaration of Helsinki. We will provide potential PAB members with written information about being a PAB member alongside contact details for the public contribution coordinators and PI. We will collect separate informed consent from all PAB members for participation at workshops and steering group meetings, discussions at workshops being audio-recorded, and public contribution activities at workshops and meetings being logged; and participation in semi-structured interviews. We will ask public contribution coordinators to provide consent for participation in semi-structured interviews. All interviews will be audio-recorded and transcribed verbatim. PAB members will be free to withdraw at any time. All information collected (e.g., background questionnaires, impact logs, audio tapes of workshop discussions, and audio tapes and transcripts of semi-structured interviews) will be processed in accordance with the General Data Protection Regulation (EU 2016/679), kept in locked fireproof cabinets, and/or stored on secure Uppsala University servers.

## Discussion

To the best of our knowledge, this is the first study to examine the acceptability, feasibility, and perceived impact of public contribution in a clinical trial in Sweden. Currently, there is no published Swedish study evaluating the impact of public contribution in research, however some Swedish studies have explored experiences of public contribution in research [58–61]. Further, there is currently a lack of public contribution in research in the oncology field especially outside of Canada, the UK, and the USA [62].

Workshops will be held online. This may facilitate the contribution of parents across Sweden and help overcome barriers to contribution potentially experienced by parent public contributors such as lack of time and working schedules [63]. However, the conduct of public contribution activities via digital meetings may limit spontaneous interactions and dynamic discussions, reducing non-verbal cues, and may result in contributors sharing less information than in face-to-face interactions [44]. To overcome these potential challenges, careful meeting planning and preparation, setting ground rules, and skillful chairing of online workshops will be essential [44]. Whilst we aim to recruit PAB members with varied backgrounds regarding age, gender, education level, and location lived in, adopting an advisory board approach to public contribution may result in recruiting parents with higher education levels who are comfortable with more of a “business approach” to public contribution [64]. As such, parents with limited Swedish and complex health and social care needs, may be excluded [65]. Further, EJDeR is developed for a Swedish-speaking population and has not yet been translated and culturally adapted for groups who speak official Swedish minority languages (i.e., Sami) or other commonly spoken languages (i.e., Arabic). Consequently, we will only include Swedish speaking PAB members whereas parents from ethnic minority groups may be excluded. An additional limitation is not involving public contributors in the development and planning of this protocol. However, public contributors have contributed to developing associated materials (i.e., advertisements, written information, consent forms, and interview guides). We will throughout the lifecycle of the CHANGE trial be open and flexible to adapting public contribution activities in collaboration with PAB members.

We hope our planned public contribution activities and the examination of the acceptability, feasibility, and perceived impact of these activities will contribute to the growing evidence of how to embed and evaluate public contribution in healthcare research. This is of particular importance given: (1) the current lack of public contribution in Swedish healthcare research and in the oncology field [62]; (2) the need to strengthen the evidence base concerning the impact of public contribution in research [19, 62, 66]; and (3) the need to enhance researcher understanding concerning how to best implement public contribution in research. We hope that adding public contribution to the CHANGE trial will provide other researchers with guidance on how to embed public contribution in clinical research and add to the growing evidence base concerning the impact of public contribution in research.

### Supplementary Information

**Additional file 1:** Guidance for reporting involvement of patients and the public checklist.

## Data Availability

As a study protocol data sharing is not applicable as no new data has been collected or processed. The datasets that will be generated and/or analyzed will not be made publicly available due to privacy or ethical restrictions but will be available from the corresponding author on reasonable request.

## Abbreviations

CBT: Cognitive Behavioral Therapy
GAD: Generalized Anxiety Disorder
GRIPP2: Guidance for Reporting Involvement of Patients and the Public Checklist
LICBT: Low-intensity Cognitive Behavioral Therapy
MRC: Medical Research Council
NIHR: National Institute for Health Research
PAB: Parent Advisory Board
PI: Principal investigator
PRP: Parent Research Partner
RCT: Randomized Controlled Trial
SMS: Short Message Service
SRQR: Standards for Reporting Qualitative Research checklist
TAU: Treatment as usual
UK: United Kingdom
